# The influence of perceived neighborhood disorder on HIV care-related decisions: A qualitative study

**DOI:** 10.1371/journal.pone.0322994

**Published:** 2025-04-30

**Authors:** Linda Jepkoech Kimaru, Priscilla Magrath, ChengCheng Hu, Sudha Nagalingam, Elizabeth Connick, Kacey Ernst, John Ehiri

**Affiliations:** 1 Department of Health Promotion Sciences, The University of Arizona, Tucson Arizona, United States of America; 2 Department of Epidemiology and Biostatistics, The University of Arizona, Tucson Arizona, United States of America; 3 El Rio Special Immunology Associates, Tucson Arizona, United States of America,; 4 Department of Medicine, The University of Arizona, Tucson Arizona, United States of America; Swiss Paraplegic Research, SWITZERLAND

## Abstract

The study used a qualitative approach to explore how perceived neighborhood disorder influences health-related decisions among people living with HIV. Recognizing the crucial role environmental factors play in health behaviors, this research seeks to bridge a gap in understanding how neighborhood dynamics affect individuals with HIV. A qualitative research design with interpretive qualitative analysis was used. The interview guide and analysis were guided by the Broken Windows Theory and Social Cognitive Theory, enabling an exploration of the intersection between environmental perceptions and healthcare behaviors. Data were collected through telephonic in-depth interviews with 18 participants from two HIV clinics from June 2022 to February 2023. Interviews were analyzed using the Dedoose software 9.0.17 and narratives were enriched using their survey data from a cross-sectional study with a validated scale to measure perceived neighborhood disorder. Our findings show that perceived neighborhood disorder influences HIV care-related decisions through a diminished sense of control pathway. Also, healthcare settings emerge as a mitigator of the influence of perceived neighborhood disorder on HIV care-related decisions by offering a sense of control. Perception of lower neighborhood disorder correlates with a strong sense of control and a preference for specialized care. As the perception of neighborhood disorder increases, there is a shift toward care settings that balance specialized services with a supportive care environment. A higher perception of neighborhood disorder leads to prioritized care settings that provide a sense of community support, and discretion, reflecting adaptations to a compromised sense of control. This research underscores the influence of neighborhood disorder on health-related decisions through the pathway of self-control, emphasizing the role that healthcare environments play as mitigators. For chronic disease management, such as with HIV, the development of healthcare settings that reinforce patient autonomy and control, alongside community efforts to diminish signs of disorder, and their underlying causes is crucial.

## Introduction

Imagine two scenarios: Person A, walking through their neighborhood where graffiti adorns the walls, buildings have deteriorated, litter scatters the sidewalks, and there is open social disorder; and Person B, residing in a neighborhood where walls are clean or intentionally decorated, buildings are well-maintained, streets are clean, and nefarious behavior is rare. These contrasting settings may shape Person A’s and Person B’s perceptions and may influence their decisions related to healthcare. This study explores the influence of perceived neighborhood order—or disorder—on healthcare-related decisions, using the interdisciplinary theoretical lens of the Broken Windows Theory (BWT) and Social Cognitive Theory (SCT). The BWT, originally a criminology concept developed by James Q. Wilson and George Kelling, suggests that minor signs of disorder and neglect, if unaddressed, create an environment where larger crimes feel permissible, leading to a snowball effect of increasing disorder [1]. Complementing this perspective, Albert Bandura’s SCT provides a robust framework for understanding behavior as a product of the dynamic interplay between personal factors, behavioral patterns, and environmental influences [2]. Central to the SCT is the concept of self-efficacy, an individual’s belief in one’s ability to control and execute the actions necessary to manage and achieve specific performance outcomes [3]. The contrasts between the environments of Person A and Person B offer settings where the concepts presented by the BWT and SCT can be explored. Examining how one’s environment can influence health behaviors and decisions through the interplay of personal experiences, actions of others, and environmental cues. SCT suggests that behaviors are influenced by a triadic interaction of personal beliefs, environmental factors, and behavior itself, emphasizing the significance of observational learning, self-efficacy, and reinforcements in behavioral formation. Theoretically, for Person A, the disordered environment may reduce self-efficacy concerning health-promoting behaviors and decisions, offering few positive role models or reinforcements. Conversely, Person B, in a more orderly setting, may have higher self-efficacy and access to models of positive health behavior and decisions. In essence, this study explores the pathways through which perceived neighborhood order/disorder as an environmental cue influences self-efficacy and thus impacts health-related decisions, as shown in **S1 Fig 1**.

We explore the pathways depicted in **S1 Fig 1** among individuals living with Human Immunodeficiency Virus (HIV), which remains a global health concern with far-reaching implications for individuals and communities [4]. Effective management of HIV is best understood through the lens of the HIV care continuum, which encompasses several key stages: initial diagnosis, linkage to care, receipt of care, retention in HIV care, antiretroviral therapy (ART) initiation, and finally, achieving viral suppression [5]. For example, being retained in HIV care is crucial, as it influences subsequent stages like ART adherence. Successful adherence to ART not only depends on initial access to medication but also requires continuous engagement and retention in HIV care thereby improving the individual’s quality of life and reducing the risk of HIV transmission to others [6]. Numerous studies have explored how various neighborhood characteristics correlate with HIV outcomes, such as risk behaviors [7], late-stage diagnosis, linkage to HIV care, and even engagement in care [8]. These associations have been primarily attributed to neighborhood-level socio-economic disparities, demographic composition, social cohesion, and neighborhood dynamic perceptions or attitudes surrounding HIV [9,10]. When it comes to neighborhood disorder specifically, studies in a meta-analysis showed that neighborhood disorder impacts health outcomes such as stress, depression, substance abuse, and overall self-reported health [11]. In healthcare settings, studies have found both social and physical signs of disorders in hospitals were positively related to staff burnout and negatively related to job satisfaction and patient safety with collective efficacy mediating the relationship [12]. Additionally, another study highlighted that unit-level dysfunction in healthcare settings can lead to normalized violations, escalating from helpful practice adjustments to conditions that contribute to patient harm [13]. However, it’s not enough to know that there is a connection; we need to understand the how and why. These are the processes in which social determinants of health influence health outcomes. Thus, a gap persists in the literature when it comes to the nuanced influence of neighborhood characteristics on health-related decision-making processes. While there’s evidence illustrating neighborhood characteristics’ associations with broad HIV outcomes, fewer studies investigate the specific ways in which these environments impact an individual’s engagement in aspects related to the continuum of HIV care. Such an understanding is pivotal, as it can illuminate tangible and intangible barriers within communities that impact optimal health behavior and outcomes. The objective of this study is to understand the influence perceived neighborhood disorder has on self-efficacy and HIV care-related decisions. Thus, our research question is “*How does the perception of one’s neighborhood—as disordered or otherwise—interact with decision-making related to HIV care?”*

## Methods

### Qualitative approach and research paradigm

This study employed a qualitative approach to explore the impact of perceived neighborhood disorder on healthcare-related decisions among people living with HIV (PLWH). Guided by the Broken Windows Theory and Social Cognitive Theory, the research was situated within an interpretivist paradigm. This paradigm was chosen to understand how individuals interpret their experiences in their neighborhoods and make sense of them, emphasizing the nuances of meaning-making inherent in interpretive qualitative research.

### Context

The study was conducted in southern Arizona, involving PLWH attending two HIV clinics. The selection of these settings was informed by their relevance to the research questions, particularly regarding the role of perceived neighborhood disorder in engagement in HIV care.

### Sampling strategy

Participants were recruited from a prior cross-sectional survey of 188 individuals examining the role of perceived neighborhood disorder in ART adherence and HIV viral suppression. A purposive sampling approach was used to recruit participants from the prior cross-sectional survey. This strategy was part of the study design to gain deeper qualitative insights from individuals who had already provided the prior data on the cross-sectional survey. Those who expressed interest in further discussing neighborhood influences on HIV care engagement were contacted via telephone to confirm their willingness to participate and schedule an interview. All interviews were also conducted via telephone. Eligible participants were 18 years or older, living with HIV, receiving care at one of two participating HIV clinics, and had completed the prior survey. The survey included PLWH of all genders who had been on ART for at least four months, had a viral load result within the past 12 months (at least four months post-ART initiation), and had resided in Arizona for at least 12 months. Exclusion criteria included inability to provide consent, conduct the interview in English, or having incomplete survey data preventing appropriate categorization of perceived neighborhood disorder. The recruitment and interviewing process spanned from June 1, 2022, to February 6, 2023. The sampling strategy aimed to reach a point of saturation where no new themes emerged from the interviews.

### Ethics statement

This study was approved by the Institutional Review Board (IRB) at the University of Arizona, Tucson (IRB number: STUDY00000098) on March 18, 2022. Each participant provided electronic written consent before participation via REDCap, and confidentiality was maintained by de-identifying interview transcripts.

### Researcher characteristics and reflexivity

As a researcher applying theoretical frameworks to explore the impact of perceived neighborhood disorder on healthcare decisions among people living with HIV (PLWH), my experience in the field of HIV/AIDS guides my inquiry. In analyzing the data, the Broken Windows Theory served as a critical lens, shaping the thematic review. My base in southern Arizona, coupled with an understanding of the healthcare settings available for PLWH in this region, informs the context and sensitivity of this research. I was also a part of the analysis in the cross-sectional survey in which these participants were drawn. Through this reflexive process, I acknowledge how my perspectives and experiences may intersect with the collection, analysis, and interpretation of data. This approach is aimed at achieving a nuanced comprehension of the participants’ lived experiences, ensuring that the research remains grounded in the realities of those it seeks to understand.

### Data collection methods

Data were collected through in-depth telephonic interviews due to pandemic-related safety concerns. Each interview lasted approximately 30 minutes to 1 hour. All interviews were conducted by Linda Jepkoech Kimaru, a researcher with training in qualitative research methods, in-depth interviewing, and HIV-related health studies.

### Data collection instruments and technologies

The in-depth interviews were conducted using a guide with probes used to generate more discussions. Participants who completed the in-depth interview were compensated with a $10 gift card.

### Units of study

Eighteen participants completed the in-depth interviews, providing varied insights into the study’s research questions. The level of participation and engagement of these individuals was consistent across all participants.

### Data processing

The interviews were transcribed verbatim and managed using Dedoose Version 9.0.17 for data coding and thematic analysis.

### Data analysis and reporting

Data analysis involved an interpretive qualitative analysis approach [14], with thematic analysis conducted in a six-phase process: data familiarization, code generation, identifying themes, reviewing themes, defining theme significance and reporting [15]. After transcription, data familiarization was conducted through multiple readings. Inductive coding was then conducted using Dedoose (Version 9.0.17), where key concepts were identified and assigned descriptive codes. These codes were grouped into preliminary themes, which were iteratively refined to ensure consistency and alignment with the study’s theoretical framework. To strengthen interpretation, participant narratives were categorized based on their perceived neighborhood disorder scores from the cross-sectional survey, allowing for a contextual understanding. Final themes were clearly defined and named before being structured into a narrative format, supported by illustrative participant quotes and a summary table to present thematic relationships. This approach aimed to capture individuals’ perceptions and experiences, aligning with the goals of interpretive qualitative research. Data saturation was reached by the 12th interview, as no new themes emerged in ongoing analysis. Six additional interviews were conducted to confirm saturation.

### Techniques to enhance trustworthiness

When we familiarized ourselves with the data, even though the participants did not use explicit words such as order or disorder to describe their neighborhoods. We noticed that the words being used to describe the neighborhoods mirrored the definitions of the Perceived or Observed Neighborhood Observation validated scale [16] that was used in the cross-sectional survey. The understanding of the cross-sectional data allowed for the contextualizing of the data in this qualitative study. Therefore, we used the 18 participants’ responses to the cross-sectional survey to categorize their narratives in the qualitative data into three categories: No perceived disorder (Score 0), Moderate perceived disorder (Score >0 and < 2.5), and High perceived disorder (Score >2.5 and < 5) as seen in Table 2. On the scale, perceived neighborhood disorder was defined as conditions and activities that residents perceive to be cues or signs of the breakdown of social control with three sub-factors, physical disorder, physical decay, and social disorder. This was a 5-point scale where 0 is not present and 5 is highly present.

## Results

According to Table 1, the median age of the participants was 54.5 years, ranging from 22 to 70 years old. Of the 18 interviewed, the majority of the participants were male 13 (72%); White 11 (61%); non-Hispanic 14 (78%); had a high school education or more 16 (88%); employed, retried or on disability 14 (78%); and 10 (56%) reported a household income of less than $25,000. The median length of stay of participants in the current zip code was 4 years with a range of 1–30 years.

**Table 1:** Participant Demographics – Total N = 18.

	n (%)
Age in years (Median [Range])	54.5(22-70)
Gender	
Female	5 (28%)
Male	13 (72%)
Race	
White	11 (61%)
Non-White	7 (39%)
Ethnicity	
Non-Hispanic	14 (78%)
Hispanic	3 (17%)
Prefer not to answer	1 (6%)
Education	
Less than high school	1 (6%)
High school	8 (44%)
More than high school	8 (44%)
Prefer not to answer	1 (6%)
Employment	
Unemployed	3 (17%)
Employed	7 (39%)
Retired/disability	7 (39%)
Prefer not to answer	1 (6%)
Household income	
<$25,000	10 (56%)
$25-50,000	3 (16%)
>$51,000	4 (22%)
Prefer not to answer	1 (6%)
Length of stay at zip code in years (Median [Range])	4 (1-30)

### Perceived neighborhood disorder spectrum with a central theme of control

A key pathway that emerged from the data was the importance of having a sense of control as a potential link through which environmental cues exert influence on decisions related to HIV care. The narratives suggest that participants felt most in control when no disorder was observed, moderately in control with moderate presence of disorder, and least in control when a high presence of disorder was noticed (see **S2 Fig 2**). First, we discuss each category of neighborhood disorder and how control is central.

#### Physical disorder.

Evaluated by the observance of cigarettes, trash, graffiti, empty bottles, etc. in their neighborhood. The 2 participants who did not perceive physical disorder in their environment expressed a well-controlled environment. For example, one participant emphasized the cleanliness and rarity of disorderly elements which speaks to a sense of predictability in the environment. “*Where I live especially? Uh... It’s pristine. There’s no graffiti, there’s no trash. There’s very little trash on the sidewalks, which is blown around on... on trash collection day. But It’s very... it’s very rare to see a piece of trash on the street. Um, [unintelligible] occasional piece of paper flying by or something, um... if that’s what you’re talking about*.” (67 y/o male, participant 321). This also speaks to the effort in community structures that control the presence of these elements in their environment. The 7 participants who noticed a moderate level of physical disorder in their neighborhoods frequently reported a compromised sense of control, especially in areas that are generally well-kept. One participant, for instance, underscored their feelings of reduced control attributed to the “*renters*” in the vicinity. “*Renters*” are commonly viewed as having a diminished dedication to the community’s collective welfare. Others felt a general sentiment that while the areas described are notin extreme disarray or featuring overtly obvious signs of neglect, there are still notable indicators of disorder. Thus, recognizing the disorder but without it seeming to reach levels of concern or distress for them. The 9 participants who noticed a high level of disorder in their neighborhoods mentioned the presence of graffiti, trash, and derogatory signage. Such an environment could diminish residents’ sense of control. For instance, statements like “*nothing is being done about it*” (60 y/o male, participant 121) reflect a feeling of helplessness and a diminished sense of control over their immediate surroundings.

#### Physical decay.

Evaluated by the observance of vacant, abandoned, vandalized, or deteriorated structures and spaces. The 3 participants noting no perceived presence of physical decay often mentioned their neighborhoods having proactive measures, such as homeowners’ associations, and felt a stronger sense of control over their environment. This is evident from the statement about proactive measures to prevent deterioration. “*And there’s, you know, if your house is starting to show, the homeowners association sends you a letter saying that it’s time for you to paint your house before things get ugly. That sort of place.*” (67 y/o male, participant 321). Such systems and regulations help to ensure a certain standard is maintained and indirectly promote a sense of pride and commitment among residents and can also be indicative of a community with strong governance and community involvement. The 9 participants noting a moderate presence of physical decay appear to be somewhat tolerant or have grown accustomed to minor wear and tear. The mention of some areas needing upkeep while others are in good shape shows a level of acceptance of the environment’s imperfections. For example, one participant said “*They kind of need, you know, a little bit of repair here and there. You know, nothing trashy I mean... uhh... they could use paint or... you know … a little bit of yard work, or stuff like that but not bad.*” (52 y/o male, participant 015). While there’s an acknowledgment of the need for upkeep, it does not seem to cause significant distress, and the decay has not escalated to a point of concern. The 6 participants noting the high presence of physical decay appear to be resigned to wear and tear due to “*low-income*” status of the neighborhood suggesting the status normalizes tolerance of physical decay. However, for some others, the deterioration translates to risk in their daily activities as it is not just an aesthetic issue but a functional and safety concern. Concerning potential risks, one participant mentions uneven sidewalk surfaces, and missing segments, posing potential hazards because they do not cater to safe mobility for pedestrians, cyclists, or those using scooters. Additionally, the participant expressed: “*They need to be redone. But I know the city is not going to do that because that would cost way too much money.*” (54 y/o male, participant 342). This suggests a perceived lack of attention from local authorities and diminished control over the issue. Another mentioned being near a dumpster, a fire hazard that poses also a risk to surrounding structures and can result in property damage.

#### Social disorder.

Evaluated by observing loitering, public intoxication, gang activities, public confrontations, and drug-related transactions in their neighborhoods, which often is linked with perceptions of safety and cohesion in the community. The 4 participants who observed no social disorder in their neighborhoods frequently attributed their sense of safety and order to several factors: an established security infrastructure, the benefit of geographical isolation, and a homogeneous community demographic. This translates to a heightened sense of control over potential social disruptions in their environment. Illustrative quotes include: “*They have security that drives around in their little... go carts” (67 y/o male, participant 321).“There, there aren’t a lot of um... nefarious type people, if you know what I mean, it’s it’s like…umm… everybody here is elderly*” (61 y/o, male, participant 012).“*The fact that we’re... all the way out here. So no, nobody messes with this area.*” (46 y/o female, no social disorder, participant 061). The 8 participants who observed moderate social disorder expressed a mix of comfort and caution regarding their surroundings and thus displayed a nuanced perception of safety, balancing both caution and comfort. They expressed an awareness of potential threats, such as crime or disruptive neighbors, yet simultaneously emphasized elements that provide them with a sense of control and safety. For some, personal measures, like having dogs or knowing the community, play a pivotal role in fostering this sense of safety. Others find solace in familiar routines, like walking at specific times or relying on personal networks and the quick response of authorities. Evidenced by statements like “*I think the police responded pretty quickly*” (58 y/o female, participant 189) or “*I have two dogs. They run the yard. The whole place is fenced in, so nobody’s going to come in here*” (61 y/o female, participant 336). Furthermore, there is a recurring theme of individuals navigating their environment with vigilance and adaptation, actively discerning which elements demand caution and which can be approached with a more relaxed attitude. This ability to differentiate and adjust, rooted in a blend of personal experiences and community insights, gives them a heightened sense of control despite the challenges presented by their surroundings. The 6 participants who observed high social disorder frequently noted overt drug use, hate crimes, break-ins, robbery, and hearing gunfire. These observances were described in an innocuous tone, suggesting normalization. There is also a reliance on their intuition, past experiences, or self-protective behaviors rather than institutional interventions (like the police), suggesting a diminished faith in these institutions. Statements like “ *I’m fairly confident in my ability to avoid dangerous situations.,” (60 y/o male, participant 042) and “the police just drive right by, they could care less, you know.*” (60 y/o male, participant 121) shows an adaptation to the disorder, but also highlights an erosion of trust in official systems. Despite the challenges, there is also an underlying theme of resilience and community identity. Some individuals in this category speak fondly of their neighborhoods or highlight the presence of “*lovely people*.” This dual perception speaks to the complex relationship individuals can have with their surroundings, finding community even in disorder. The narratives illustrate a challenge to participants’ sense of control in their surroundings rooted in both the tangible (like crime or neglect) and intangible (like feelings of vulnerability or perceived neglect from authorities).

### Influence of perceived neighborhood disorder on HIV care-related decisions

Participants perceiving no disorder in their neighborhoods were drawn primarily to healthcare expertise as shown in **S3 Fig 3**. Their surroundings did not impose a pressing need for a specific care environment, allowing them to prioritize specialized care. Their environment does not push them to seek care; rather, they are pulled by specialized care. This was illustrated by one participant saying “*Well, I have a dozen different specialists that I see, um... my primary care physician is basically unavailable. But I have established relationships with, you know... um, I have a neurologist, gastroenterologist podiatry, nephrology... I mean, you name it. Uh, cardiology, vascular surgeon, uh... lung doctor and I’ve, I’ve got a urologist. So. It’s like, you know, I’ve seen all... all those dermatologists, HIV doctor... so I’m seeing almost all of them on a regular basis, you know, for routine maintenance.*” (67 y/o male, participant 321).

Participants perceiving moderate disorder in their neighborhoods expressed a nuanced balance between specialized care/services and the healthcare environment, just as they felt moderate in control of their environment. They prioritize a sense of choice and control in seeking HIV care. They may weigh options and select facilities that offer a blend of specialized care/services and a conducive environment. For instance, one participant candidly shared “*And then, but there’s also ____ [hospital]. ______[hospital], that I guess I could go to, I actually don’t. I only live probably half a mile from the... Less than 1/2 a mile from __________[hospital], but I don’t go there. because I and [inaudible]. There when I when I used to go there for like HIV and all that, and I just didn’t like the way that they ran it. It just it wasn’t. I forget. It was just like… Scheduling things with… because it’s not easy. So, I really like _____[CHC] because it was more kind of personal like you know, people and interactions where it wasn’t like a big hospital*.” (44 y/o male, participant 020). In the backdrop of a chaotic environment, having a one-stop shop or integrated comprehensive service can become more than just a place for medical care. They stand out as havens of security, understanding, and refuge, sharply contrasting the unpredictability of their residential settings. Many participants highlighted the value of having an integrated service model. One participant expressed this sentiment “*I love the fact that everything is… my behavioral health, my medical health, and my therapy are all in one place. And so those doctors all communicate with each other very well.*” (58 y/o, female, participant 189).

Participants perceiving high disorder in their neighborhoods felt the least sense of control and prioritized healthcare facilities where they felt listened to and cared for, even if these facilities were not the most convenient suggesting an unmet need for support and understanding in their immediate environments. Although most expressed the same sentiments regarding the preference for a one-stop-shop in their HIV care settings, what was different was the emphasis on the quality of treatment they received and the logistical challenges of getting to the care. Participants perceiving high disorder in their neighborhood seemed to seek its opposite in their HIV care. Their language often mirrored their feelings about their neighborhoods, phrases like “*they care less*” shifted to “*they care*” when describing preferred healthcare institutions. For instance, one participant captured this sentiment by saying “*The hospital um, I’ve, I’ve been to both. My old one is ____[CHC] which is really far away, and more on the east side. Which is inconvenient for me to go to. Really friendly, really cared for you. It was just too much. As the other one, the hospital. Is a more convenient. But you get lost as a number*.” (60 y/o male, participant 121). Some in high-disorder neighborhoods also expressed transportation challenges and their narratives revealed a pressing need to strategize and become more efficient, craving some semblance of predictability in their mobility. For example, one participant expressed: “*Before I moved to this neighborhood I was living on the ____ before and so, it. It was. It was much. It was more, more difficult than to come to an appointment because it was almost… sometimes it might be like a two-hour bus ride. And I think I think now it’s. You know, I just take two buses and I’m there. So, I think it’s a lot easier now. To get care for my HIV.*” (60 y/o male, participant 042).

### Healthcare setting mitigation

Healthcare settings seem to be a mitigating factor on the influence of environmental cues through a sense of control pathway when making HIV care related decisions as shown in **S4 Fig 4**.

The role of healthcare settings is illustrated by comparing responses from participants in moderate and high neighborhood disorder categories. For those perceiving moderate disorder, their sense of control emanates from their ability to choose integrated services, have personalized care experiences, and benefit from digital communication tools. They seem to have the bandwidth to prioritize these aspects of their healthcare journey because their external environment may not be as chaotic or demanding. When asked about recommendations in making seeking care better, participants perceiving moderate disorder highlighted support systems (support groups to address isolation), consistent healthcare personnel (minimized changes in healthcare personnel), and transportation support from healthcare settings. When looking at healthcare preferences and qualities of care through the lens of a ‘sense of control’ it becomes clear that those who perceive moderate disorder lean towards healthcare solutions that are:

1) Predictable - opting for healthcare settings that offer integrated services, structured routines, and digital communication tools. This is illustrated by participants stating, “*I also like the fact that they have an excellent patient portal online, so I can just go in there anytime they want and look at my own labs and I am smart enough.*” (58 y/o, female, participant 189) and “*I find that having the doctors in the pharmacy in the same building kind of facilitates moving things, a little bit quicker*” (70 y/o male, participant 124)
2) Autonomy – preference for personalized care, convenient medication delivery systems, and affordable care options. Some statements to this effect include: “*I go to ____ and I get my three-month supply and yeah, that’s pretty much it nothing too complicated. Everything is very simple…. they use a sliding scale fee, you know, for their services. So, you don’t have to have insurance*.” (22 y/o male, participant 323)
3) Community-centric – seeking out healthcare settings with familiar faces and the availability of support groups. This is evidenced by participants noting “*You know, I knew the nurse assistant and... it felt more like a community…. I didn’t feel like I was going into this place where each time I went in, I was like a brand-new stranger*.” (44 y/o male, participant 020)

In contrast, participants perceiving high disorder focused on creating a semblance of order and predictability in navigating their environment getting to care and the environment of care, rather than the services they receive. When asked for recommendations in making seeking care easier they highlighted safe transportation (better lighting when traveling in the evening from care; appointment timing to avoid traveling at unsafe hours) and transportation improvements increasing the frequency of bus services to enhance navigation to health facilities. Their choices seem to be driven by a need to manage the immediate challenges posed by their environment, although they seemed to prefer the integrated services just as those who were in moderately disordered neighborhoods. Individuals navigating perceiving high disorder gravitate towards healthcare settings that foster community and belonging, offer robust logistical support to ensure consistent care access, and maintain utmost discretion:

1) Community-centric – settings that have social support systems, “*I belong to a program called ____ at ____[CHC]. And what it is, is it started out as people that are HIV positive get together once a week. We get to fill our med boxes with the pharmacist there as a group and then we get lunch and then we have a guest lecturer come in and do a lecture on HIV.*”(61 y/o male, participant 117)
2) Can provide logistical support – setting offering logistical support evidenced by statements like “*The insurance transportation____,.... We have to call in our appointments... our transportation 3 days before, working days.*” (55 y/o female, participant 103) and “*they’re really good at shipping my medicine, like overnight. Since I’m not in ____ they UPS it to me, and so I mean I get it pretty quickly now*.”(54 y/o male, participant 342)
3) Discrete – settings that can foster the need for discretion “*they don’t call out the prescriptions to you so there’s nothing …. like looking at normal medication and the clinic that I go to is a great clinic and they’ve got a you know that’s where most of the HIV positive patients go and so most of us are getting goods at ____, having said that … that’s their main one most of us there are HIV so there is no problem.*” (44 y/o male, participant 001)

**Table 2** presents the themes and sub-themes in the narratives above by showing how individuals’ perceptions of neighborhood disorder may influence their healthcare-related decisions and preferences for certain characteristics of healthcare services through the pathway of sense of control. The three main themes include 1) Sense of control, 2) HIV care-seeking decisions, and 3) Preferred care characteristics, each with sub-themes that capture variations in participants’ experiences based on their level of perceived neighborhood disorder. Sense of control is examined through categories of physical disorder, physical decay, and social disorder. While HIV care-seeking decisions and preferred care characteristics highlight how participants’ healthcare choices and preferences shift depending on their neighborhood perceptions. The table systematically organizes these findings across three levels of perceived neighborhood disorder—none, moderate, and high. We summarized participant narratives from each of the subcategories, for example for the two participants who reported a score of 0 for physical disorder on the perceived neighborhood disorder scale – their narratives regarding physical disorder were themed to describe their sentiments on the sense of control within their environment.

**Table 2:** Health care related decisions by degree of disorder.

Theme	Categories/sub-categories	Sub-themes by the degree of perceived neighborhood disorder from the PNOS Scale			Total
		No perceived disorder (Score 0)	Moderate perceived disorder (Score >0 and < 2.5)	High perceived disorder (Score >2.5 and ≤ 5)	
Sense of control	Physical disorder (cigarettes, trash, and empty bottles in the street; graffiti (political or non-political)	(n = 2)	(n = 7)	(n = 9)	18
		Well-controlled environment translating to predictability in the environment.	Compromised sense of control, recognizes disorder but it does not seem to reach levels of concern	Normalized diminished sense of control reflecting feeling of helplessness	
	Physical decay (vacant, abandoned, vandalized, run-down houses and buildings; deteriorated residential and recreation places)	(n = 3)	(n = 9)	(n = 6)	18
		proactive measures to control deterioration indicative of a community with strong governance and community involvement	Somewhat tolerant or have grown accustomed to minor wear and tear as it has not escalated to a point of concern because they can be controlled	Normalized tolerance of physical decay translating to accepted risk in their daily activities	
	Social disorder (loitering, public intoxication, gangs, public fighting, and selling drugs)	(n = 4)	(n = 8)	(n = 6)	18
		Sense of safety and order due to security infrastructure, geographical isolation, and a homogeneous community translating to a heightened sense of control over potential social disruptions	A balance of both caution and comfort; aware of potential threats simultaneously emphasizing elements that provide a sense of control.	Normalized diminished sense of control and erosion of trust in institutions that control social disorder thus adopting self-protective behaviors	
Perceived neighborhood disorder scores	Includes total average scores of physical disorder, physical decay, and social disorder	(n = 1)	(n = 9)	(n = 8)	18
HIV care-seeking decisions		No pressing need for a specific care environment allowing prioritization of specialized care/services	Balanced between the need for specialized care/services and the healthcare environment.	Prioritized healthcare settings that listened to and cared for them regardless of convenience	
Preferred care characteristics		Specialized care	Predictable, autonomous, and community-centric care	Community-centric, logically supportive, and Discretion	

## Discussion

Optimal health outcomes for people living with HIV hinge on their engagement in care. Looking at what drives healthcare-related decisions among individuals requiring long-term disease management is key to improving engagement in care. Our study suggests that a ‘sense of control’ is a key potential pathway through which perceived neighborhood disorder exerts its influence on HIV care-related decisions. It also finds that healthcare settings mitigate the influence of perceived neighborhood disorder by offering services that provide an ‘assured sense of control’ as illustrated in **S4 Fig 4**. In BWT terms, the self-defined “broken windows” in the neighborhood (such as trash in the street, deteriorated surroundings, and unaddressed social disorder) serve as a poignant reflection of broader systemic issues they have encountered. Consequently, in their healthcare experiences, they gravitate towards settings that present a stark contrast, embodying order, and structure, and offering services that respond to their needs.

Our study indicates that the perception of neighborhood disorder as an environmental cue influences HIV care related choices through the pathway of diminishing sense of control (see **S1 Fig 1**). The literature supports this in a study that found that neighborhood disorder is negatively associated with a sense of personal control, in this study the association is moderated by individual characteristics such as race, socioeconomic status, and economic hardship [17]. Another study showed that neighborhood disorder significantly elevates feelings of powerlessness. Social ties with neighbors partially mediated this relationship although it remained substantial even after considering the influence of social ties, highlighting the direct impact of environmental disorder on an individual’s sense of control [18]. In addition, a study demonstrated that neighborhood disorder cultivates mistrust, compounding the sense of control [19], this dynamic heightens psychological distress, highlighting the influence of environmental disorder on personal agency. Thus, we can see how in this study participants’ preference for healthcare settings that offered services that provided them with more control and personal agency increased when their degree of perceived neighborhood disorder increased.

Our study also found a second pathway where healthcare settings mitigate the influence of perceived neighborhood disorder as an environmental cue on healthcare-related decisions. Varying degrees of perceived neighborhood disorder influenced healthcare-related decisions in that participants sought care or preferred care that provided them with a sense of control over their health counteracting their neighborhood perceived experiences. The concept of healthcare settings as a mitigator providing a sense of control (**S4 Fig 4**) is recognized in the literature by studies illustrating that healthcare environments play a crucial role in continued engagement in care. Specifically, providing this sense of control through services such as patient-friendly clinic services, including transportation assistance and reminder systems, along with positive relationships with healthcare providers, has been identified as a key factor that facilitates retention in care among people living with HIV [20,21]. Additionally, tailored healthcare models that address social and structural barriers, integrate mental health services, and offer comprehensive patient support services are vital for enhancing a sense of control and improving health outcomes in this population [20,21].

### Public health implications

The interplay of social determinants of health as reflected in neighborhood quality and individual healthcare choices, as underscored by our findings, sheds light on the complex dynamics of care engagement among people with HIV. Our study demonstrates how neighborhood disorder not only diminishes one’s sense of control but also instills a sense of powerlessness that can pervade all aspects of life, including healthcare engagement. Our study has important public health implications not just for those living with HIV but extends for any chronic disease management. Healthcare settings serve as more than mere medical centers, especially for individuals perceiving disorder in their environments. These settings can offer a counter to neighborhood disorder through being spaces of solace, understanding, and security. Our findings suggest that those designing healthcare settings should consider patients’ needs to feel in control of their health; find community in their care and its potential to facilitate engagement and self-efficacy in treatment. Ultimately, the cultivation of such environments is critical for optimal health outcomes and underscores the need for systemic approaches that encompass the broader socio-environmental context of patients’ lives. Our study’s emphasis on the importance of neighborhood and healthcare environments in providing an ‘assured sense of control’ resonates with the Blue Zones Project’s approach of effecting policy and environmental changes rather than relying solely on individual efforts [22]. The Blue Zones Project is an initiative inspired by the study of “Blue Zones,” regions around the world where people live longer than average [22]. The project seeks to enhance health and well-being in communities globally by implementing lessons from these areas such as modifying environmental factors, policies, and social networks to promote healthier lifestyles [22]. The success of the Blue Zones Project in enhancing public health outcomes through community-wide initiatives suggests that similar strategies could be employed to improve the management of diseases that require long-term management like HIV. The Blue Zones Project takes this a step further by not just repairing ‘broken windows’ but by fostering community-centric characteristics that encourage collective responsibility for health and well-being.

### Strengths and limitations

The strengths of this study are in combining the social cognitive and broken windows theory in understanding HIV care-related decisions. Using the participants’ cross-sectional survey data to categorize their neighborhood description as an additional source of information. However, the study is not without limitations. Our study recruited from set HIV clinics from a specific geographical area which may impact the transferability of findings to different populations with varying socio-economic and cultural contexts. The limited diversity in gender and ethnicity of our sample may further diminish the generalizability. The use of telephonic interviews necessitated by the pandemic restrictions may not capture non-verbal cues important for qualitative research. Lastly, the data analysis while methodologically sound, is dependent on the subjective interpretations of the researcher, despite efforts at reflexivity. The interpretive analysis may impose inherent biases, as it is influenced by the researcher’s perspectives and theoretical framings.

## Conclusion

Perception of disorder in one’s neighborhood lowers one’s sense of control which can translate to health-related decisions such as selecting a care facility. Healthcare settings can play a crucial compensatory role by providing an environment that restores this sense of control, thereby promoting health and well-being for individuals requiring consistent care. Future research should delve deeper into the ways healthcare services and community interventions can systematically reinforce a sense of control, offering the potential to improve health outcomes for those living with chronic conditions.

## Supporting information

S1 Fig 1 The influence of perceived neighborhood order/disorder on health-related decisions. (TIF)

S2 Fig 2 The influence of environmental cues on the sense of control. (TIF)

S3 Fig 3 The influence of environmental cues through the sense of control pathway on HIV care-related decision making. (TIF)

S4 Fig 4 The influence of environmental cues through the sense of control pathway is mitigated by healthcare settings. (TIF)
