# Supplementary figures and images for: The influence of perceived neighborhood disorder on HIV care-related decisions: A qualitative study
Source: PLoS One. 2025 Apr 30;20(4):e0322994. doi: 10.1371/journal.pone.0322994 (PMC12043128; doi:10.1371/journal.pone.0322994)

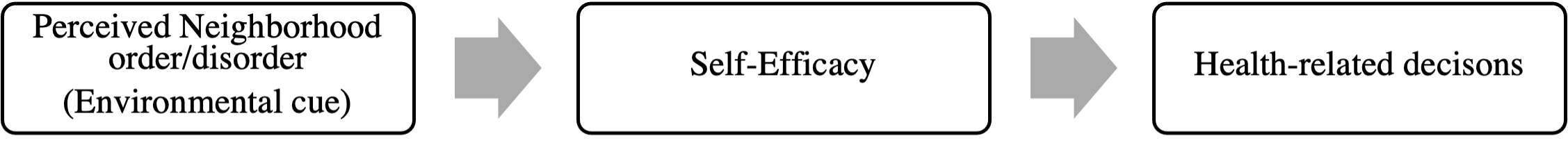

Supplement: S1 Fig 1 — (TIF) [file pone.0322994.s001.tif]

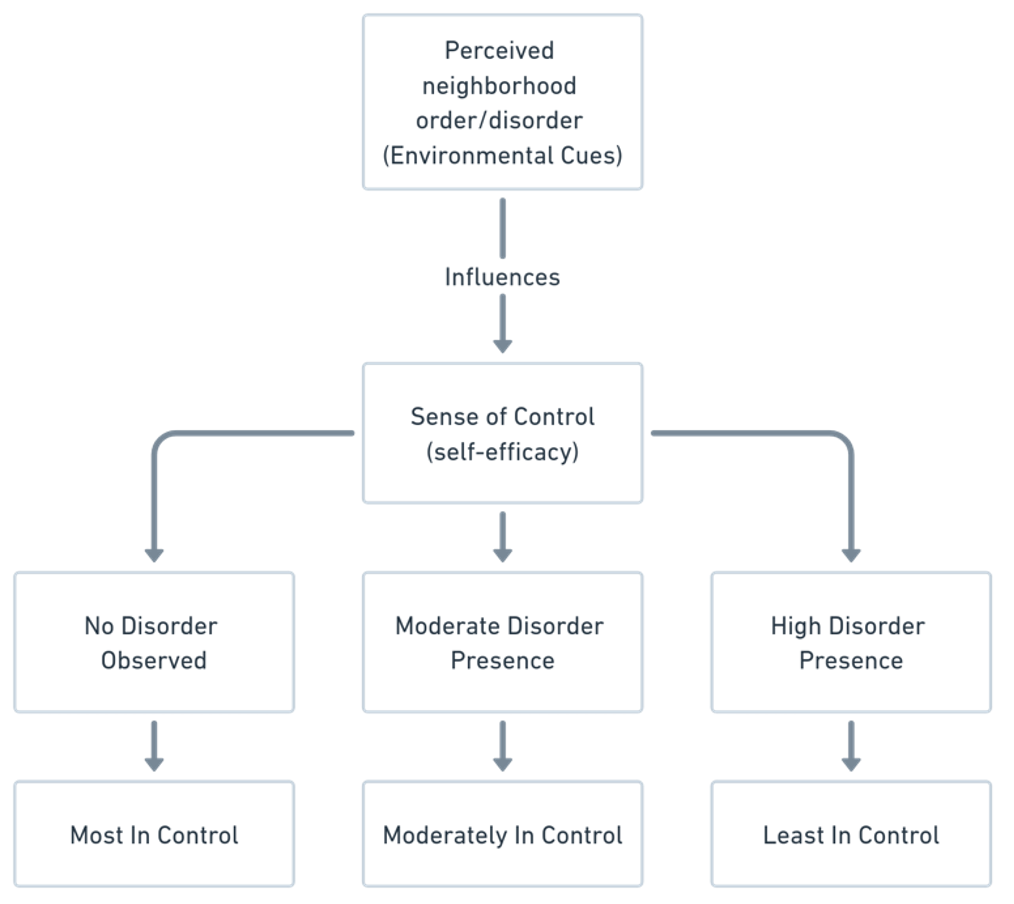

Supplement: S2 Fig 2 — (TIF) [file pone.0322994.s002.tif]

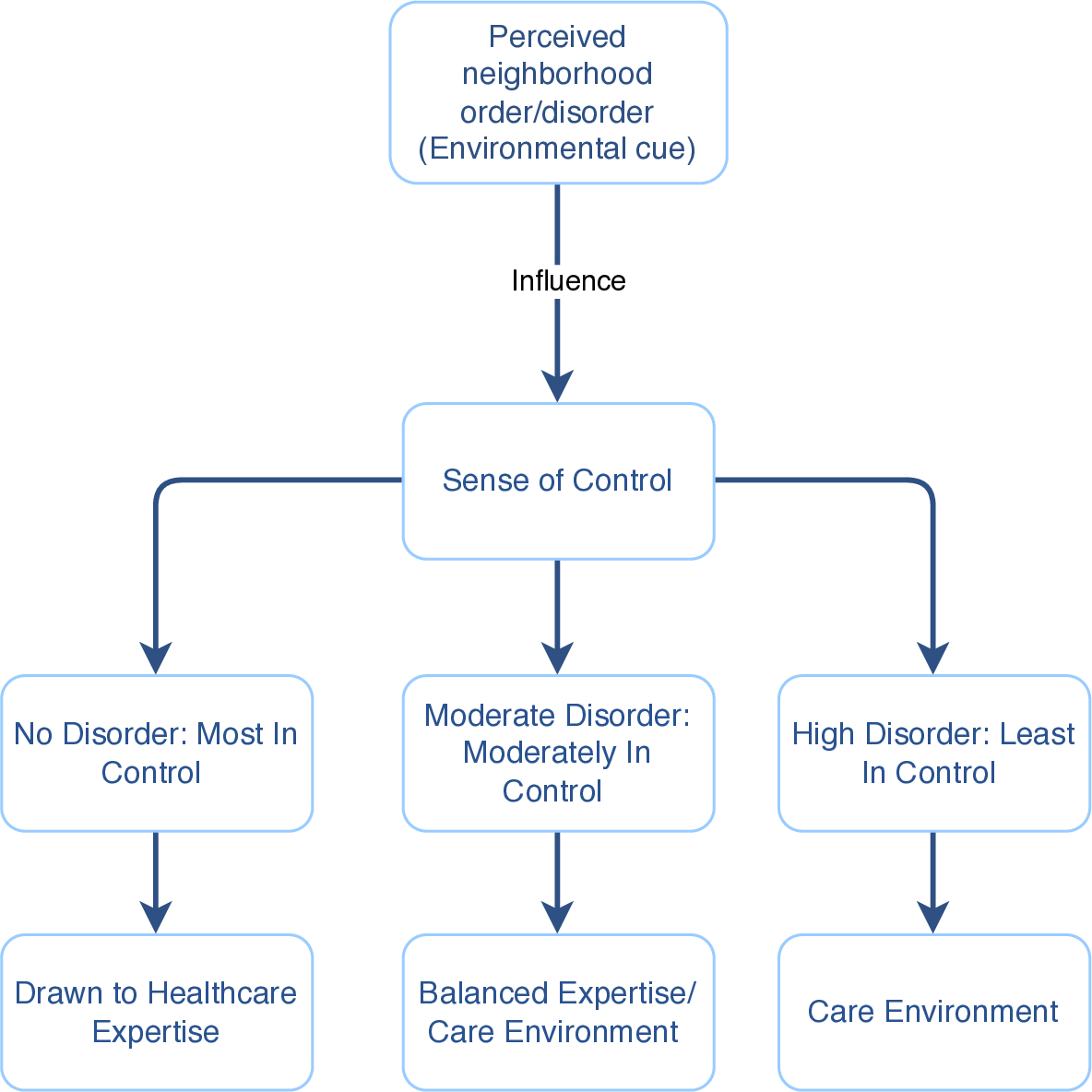

Supplement: S3 Fig 3 — (TIF) [file pone.0322994.s003.tif]

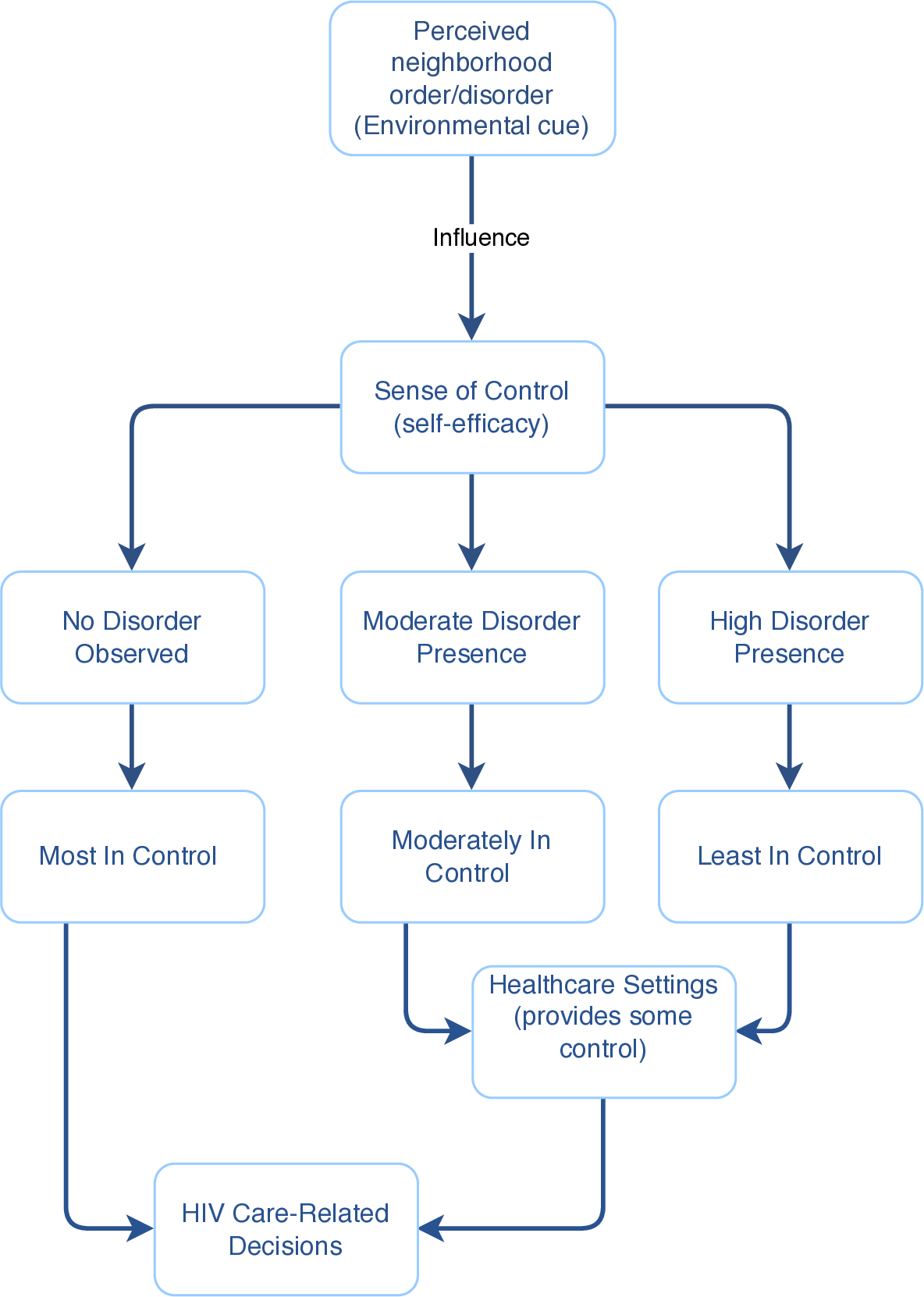

Supplement: S4 Fig 4 — (TIF) [file pone.0322994.s004.tif]
